# Turning Cucurbit[8]uril into a Supramolecular Nanoreactor for Asymmetric Catalysis

**DOI:** 10.1002/anie.201505628

**Published:** 2015-09-07

**Authors:** Lifei Zheng, Silvia Sonzini, Masyitha Ambarwati, Edina Rosta, Oren A Scherman, Andreas Herrmann

**Affiliations:** Department of Polymer Chemistry, Zernike Institute for Advanced Materials, University of Groningen Nijenborgh 4, 9747 AG Groningen (The Netherlands) E-mail: a.herrmann@rug.nl; Melville Laboratory for Polymer Synthesis, Department of Chemistry, Cambridge University Lensfield Road, Cambridge CB2 1EW (UK); Department of Chemistry, King's College London Britannia House, 7 Trinity Street, London SE1 1DB (UK)

**Keywords:** asymmetric catalysis, Diels-Alder reaction, host–guest systems, macrocycles, supramolecular chemistry

## Abstract

Chiral macromolecules have been widely used as synthetic pockets to mimic natural enzymes and promote asymmetric reactions. An achiral host, cucurbit[8]uril (CB[8]), was used for an asymmetric Lewis acid catalyzed Diels–Alder reaction. We achieved a remarkable increase in enantioselectivity and a large rate acceleration in the presence of the nanoreactor by using an amino acid as the chiral source. Mechanistic and computational studies revealed that both the amino acid–Cu^2+^ complex and the dienophile substrate are included inside the macrocyclic host cavity, suggesting that contiguity and conformational constraints are fundamental to the catalytic process and rate enhancement. These results pave the way towards new studies on asymmetric reactions catalyzed in confined achiral cavities.

Reaction pockets are considered to be responsible for the origin of enhanced rates and high selectivities in enzyme-catalyzed transformations. Although the detailed working mechanism is often unclear, stabilization of the transition state and binding of the substrate in a beneficial orientation and conformation within the pocket appear to be fundamental.[1] Inspired by nature, supramolecular systems with confined hydrophobic cavities where apolar reactants are recognized by host–guest interactions are potential candidates for the development of efficient enzyme mimetics.[2] Among them, the pumpkin-shaped family of macrocycles cucurbit[*n*]urils, CB[*n*], have been shown to promote both photochemical and thermochemical reactions, entailing rate accelerations as well as improving stereo- and chemo-selectivities.[3] However, unlike some analogues, such as cyclodextrins,[4] calixarenes,[5] and nanocages,[6] employing CB[*n*] in asymmetric catalysis remains a challenge. The main reasons are related to the intrinsic achirality of cucurbiturils and the nontrivial synthetic efforts in constructing chiral supramolecular assemblies.[7] To the best of our knowledge, only one example has been reported, showing a marginal increase of *ee* values from 16 % to 18 % when CB[8] was added to the reaction mixture.[8]

Moreover, cucurbiturils not only exhibit a hydrophobic cavity, which has been extensively applied for improving reactivity and selectivity, but they also possess two carbonyl rims with a known affinity for both organic and inorganic cations.[3, 9] Nevertheless, CB[*n*] binding to metal ions, especially for catalysis, has not been fully exploited.[3d,e]

To address these challenges, we herein present a novel design of a supramolecular CB[*n*]-based system forming a chiral nanoreactor by the combination of three components: amino acids, Cu^2+^, and CB[8]. Here, amino acids were applied as a source of chirality, and Cu^2+^ acted as a potential active site to expand the reaction scope from conventional photo- and thermo-chemical reactions to new types of transformations, such as the Lewis acid catalyzed Diels–Alder (D-A) reaction shown in Scheme 1. CB[8] can bind the chiral copper complex and further serves as the recognition site for substrates. A catalytic amount of the CB[8]-reactor was enough to achieve both rate acceleration (up to 9.5 times) and enhanced diastereoselectivity and enantioselectivity, yielding *ee* values of the products of up to 92 %. To the best of our knowledge, this is the first demonstration of achiral cucurbiturils playing an active role in substantially affecting enantioselectivity during a catalytic reaction.

**Scheme 1:**
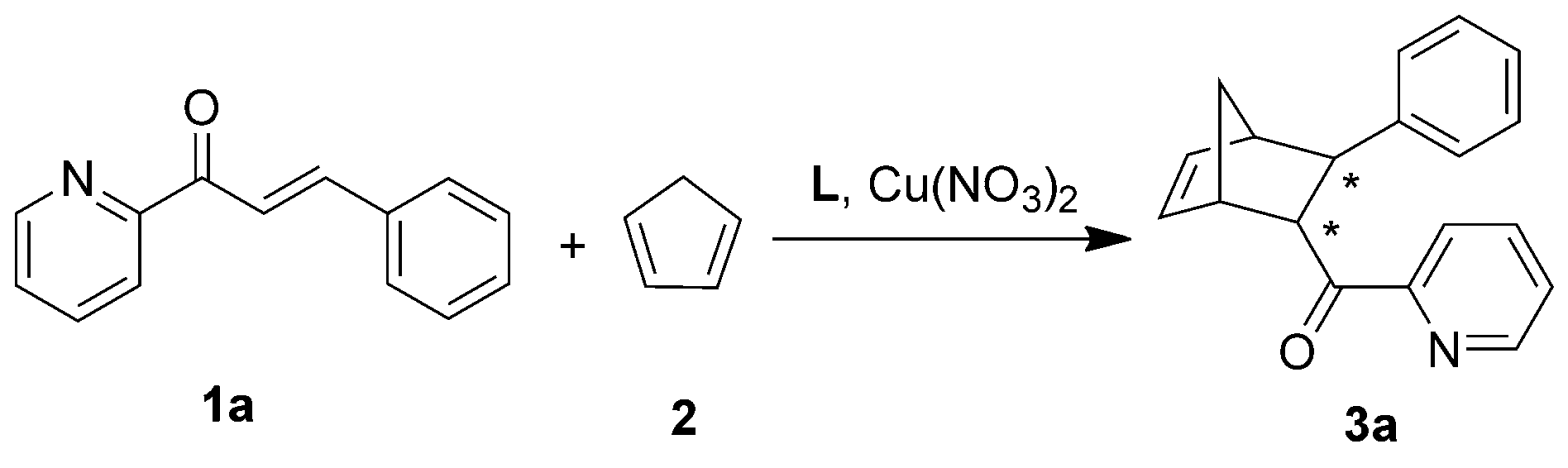
Asymmetric D-A reaction; L stands for the amino acid used as ligand to create the asymmetric copper catalyst.

Following our design strategy, we prepared the catalytic assembly using different CB[*n*], including CB[6], CB[7], and CB[8], and different amino acids, which are known to bind to specific members of the cucurbituril family.[9] As previously reported, CB[6] shows reasonable affinity towards positively charged aliphatic chains, such as those present in lysine, while CB[7] and CB[8] form inclusion complexes with aromatic amino acids (Figure 1).[9]

**Figure 1:**
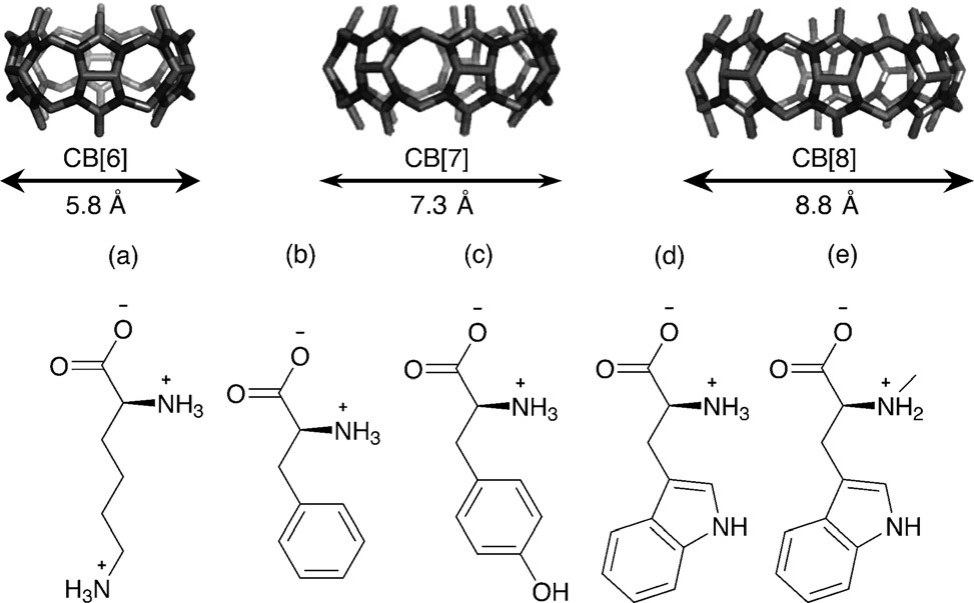
Components for the screening of the supramolecular system catalyzing the D-A reaction. Top row: CB[6], CB[7], and CB[8] as well as their outer diameters. Bottom row: a) lysine, b) phenylalanine, c) tyrosine, d) tryptophan, and e) abrine.

The three members of the CB[*n*] family were then combined with different amino acids (Figure 1, compounds **a**–**e**) and the resulting supramolecular host–guest systems were tested in the benchmark Cu^2+^-catalyzed D-A reaction of azachalcone (**1 a**) with cyclopentadiene (**2**) under optimized conditions (Supporting Information, Section S3).[10] We chose this particular reaction as a proof-of-concept because the cycloaddition was known to be highly sensitive to the presence of other macromolecules, such as DNA,[11] antibodies,[12] cyclodextrin,[13] and molecular capsules.[14] As expected, CB[*n*]⋅Cu^2+^ adducts formed racemic products, indicating that enantioselectivity only can be induced by the chiral amino acids (Table 1). Upon using lysine as a ligand alone, 2 % *ee* was achieved, and no higher *ee* values were obtained when this amino acid was combined with CB[6]. In contrast, when Cu^2+^ was complexed with amino acids bearing aromatic side chains (**b**–**e**), higher enantioselectivities (13–72 % *ee*) were obtained (Table 1). When CB[7] was added to the Cu^2+^–aromatic amino acids adducts, decreased enantioselectivities were observed compared to the pristine amino acid catalyst system. Upon addition of CB[8], nearly no effect was found for phenylalanine (**b**) and tyrosine (**c**), but, remarkably, both assemblies CB[8]⋅**d**⋅Cu^2+^ and CB[8]⋅**e**⋅Cu^2+^ gave the same product with highly increased diastereo- and enantioselectivities compared to **d** and **e** alone (Table 1). The catalyst system consisting of CB[8]⋅**e**⋅Cu^2+^ resulted in 92 % *ee*. These results show that the size of the hydrophobic cavity is a crucial factor in influencing the catalytic performance. It has to be large enough to incorporate the chiral moiety and still leave sufficient space for accommodating the substrates.[15]

**Table 1:** Enantiomeric excesses of Diels–Alder products using different copper catalyst assemblies^[a]^

Ligand		a	b	c	d	e
	–	2	13	32	31 (90:10)^[b]^	72 (90:10)^[b]^
CB [6]	0	2	–	–	–	–
CB [7]	1	–	9	10	13	55
CB [8]	0	–	12	32	**73 (96:4)**^[b]^	**92 (97:3)**^[b]^

[a] General conditions: **1 a** (1.5 μmol), **2** (4 μL), Cu^2+^ (3 % mol), **a**–**e** (4.5 % mol), CB[n] (6 % mol) in 1 mm phosphate buffer (PBS) with pH 7.4, for 24 h at 6 °C. Data were obtained from the crude product on chiral phase HPLC. The *ee* values are averaged over two experiments and are reproducible within ±2 %. [b] Endo/exo ratios are in parentheses. In all reactions, conversions determined by HPLC are >99 %. The absolute configuration of the main products were identified as the (2*R*,3*R*)-enantiomer.

To further elucidate the catalytic activity, the apparent second-order rate constants (*k*_app_) of the D-A reactions mediated by different copper complexes were measured from the initial decrease in UV absorption of **1 a** (Table 2; Supporting Information).[10] In the presence of CB[8], a modest 4-fold rate increase was found for **d**⋅Cu^2+^. A more pronounced 9.5-fold rate acceleration was observed in the case of **e**⋅Cu^2+^. The results produced by CB[8]⋅**e**⋅Cu^2+^ clearly indicate the common trend between reactivity and selectivity, namely, the most active combination also gave rise to the highest enantioselectivities.

**Table 2:** Kinetic data and isobaric activation parameters at 298 K for the Diels–Alder reactions^[a]^

	d⋅Cu^2+^	CB[8]⋅d⋅Cu^2+^	e⋅Cu^2+^	CB[8]⋅e⋅Cu^2+^
*k*_app_ (mol s^−1^)	0.022	0.089	0.050	0.48
*K*_eq_(m^−1^)	1.77×10^3^	2.07×10^4^	2.31×10^3^	1.91×10^4^
*k*_cat_ (mol s^−1^)	0.252	0.184	0.451	1.04
Δ*G*^≠^(kcal mol^−1^)	19.7	18.9	19.2	17.9
*ΔH*^≠^(kcal mol^−1^)	4.4	7.2	2.6	5.3
*T*Δ*S*^≠^(kcal mol^−1^)	−15.3	−11.7	−16.6	−12.6

[a] Fixed ratio of amino acids to Cu^2+^ 1.5:1. In the presence of CB[8], CB[8]:**d**/**e**:Cu^2+^=2:1.5:1.

Encouraged by these data, the origin of this rate acceleration was further investigated using the methods described previously by Engberts and co-workers.[16] The proposed catalytic cycle where the nanoreactor is expected to influence the rate of the D-A reaction is depicted in the Supporting Information, Scheme S1. In the first step, the reversible association between nanoreactor and dienophile generates a readily activated substrate for the reaction with the diene; then the D-A reaction occurs irreversibly. Finally, the product has to depart from the active site, regenerating the catalyst for the next cycle. In total, the overall rate of the reaction (*k*_app_) is determined by three constants: *K*_eq_, *k*_cat_, and *k*_d_. Since the kinetic measurements were performed with a large excess of catalyst, the contribution from *k*_d_ can be ignored under this condition. The equilibrium constant, *K*_eq_, was measured independently from a series of UV/Vis absorption titration experiments (Supporting Information, Figure S2). The catalytic rate constant, *k*_cat_, was then calculated according to the determined apparent rate constant (*k*_app_) and *K*_eq_ (Supporting Information, Section S4.3). As listed in Table 2, for both **d**⋅Cu^2+^ and **e**⋅Cu^2+^, *K*_eq_ in the presence of CB[8] showed an increase of around one order of magnitude; however, the *k*_cat_ does not change significantly. These results clearly show that the observed rate enhancement in the presence of CB[8] is mainly on account of an increased affinity of the dienophile to the cucurbit[8]uril–copper complex.

Next, the Eyring equation was employed for the calculation of isobaric activation parameters by measuring *k*_app_ at various temperatures (Supporting Information, Figure S3). This revealed that the enhanced rate constant in the presence of CB[8] is largely driven by the gain in entropy, which is partially compensated by a negative effect on enthalpy. This favorable effect on Δ*S*^≠^ suggests that classical hydrophobic interactions are responsible for expelling surface-bound water molecules from both the hydrophobic cavity and from the non-polar substrate, and therefore are most likely a major driving force for rate acceleration.[12b, 17]

As the experimental data suggested the formation of a supramolecular catalyst capable of binding and orienting the substrate efficiently, the nanoreactor cavity and its interaction with the diene were further investigated using a computational approach (Supporting Information, Section S5). First, the most stable conformation of the active catalytic complex was determined, resulting in the *trans*-cisoid structure (Supporting Information, Table S2 and Figure S4 a). Importantly, the same conformer was previously suggested to be the most stable.[18]

Once obtained, the stability of the complex inside the CB[8] cavity was verified. Moreover, the complex appeared to be almost completely buried inside the host, with only the vinylbenzene unit of the azachalcone protruding towards the solvent, and thus accessible for the diene approach (Supporting Information, Figure S4 b). Since these results were in full agreement with the experimental data, the relative energies and the structure of the nanoreactor in the presence of cyclopentadiene were also calculated. The structures obtained (Figure 2) showed a biased interaction between the catalytic complex and the diene: when the cyclopentadiene approached from the same side as the amino acid the complex needed to twist and open slightly, to accommodate the diene, with a subsequent energetic cost. This process, which also appeared to take place in the absence of CB[8] (Supporting Information, Figure S5 c), was even more unfavorable in the presence of the macrocycle on account of the space constraints introduced by the host cavity. The relative energies for the complex with and without CB[8] when cyclopentadiene was approaching from different directions were also calculated (Supporting Information, Table S3). As expected, approach from the same side as the amino acid was disfavored both with and without CB[8]; however, the difference in energy between diene approach from the same side versus the opposite side of the amino acid was higher in the presence of the macrocyle, namely 3.1 vs 6.5 kcal mol^−1^ (Supporting Information, Table S3). These results provided a possible explanation for the observed higher enantioselectivity in the presence of CB[8] and fully supported the hypothesis of the substrate binding to the **d**⋅Cu^2+^ complex within the host cavity.

**Figure 2:**
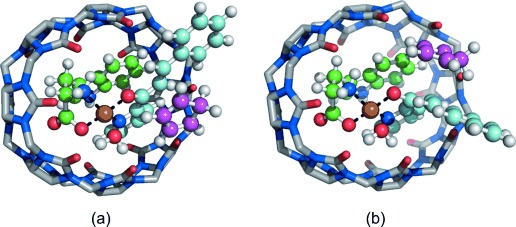
Nanoreactor–1 a complex with cyclopentadiene approaching from a) the opposite side and b) the same side of the amino acid in the endo direction.

To gain a further understanding of how the supramolecular asymmetric nanoreactor assembles, structural studies on the catalyst assembly as well as the molecular recognition between nanoreactor and dienophile were carried out with the aid of ^1^H NMR, fluorescence, and UV/Vis spectroscopies, and isothermal titration calorimetry (ITC). First, the inclusion of **d**⋅Cu^2+^ and **e**⋅Cu^2+^ within the CB[8] cavity was evaluated through ^1^H NMR (Supporting Information, Figures S7–S10). The addition of CB[8] into either a solution of **d**⋅Cu^2+^ or **e**⋅Cu^2+^ resulted in an upfield shift of the aromatic signals, which corroborated the inclusion of the indole ring inside the hydrophobic cavity of the macrocycle. Further evidence of binding was obtained from fluorescence measurements, where both fluorescence quenching as well as a distinct blue-shift (ca. 20 nm) were observed upon addition of CB[8] to **d**⋅Cu^2+^ or **e**⋅Cu^2+^ solutions (Supporting Information, Figure S12). Moreover, ITC experiments demonstrated the presence of a binding event between **d**⋅Cu^2+^ or **e**⋅Cu^2+^ and CB[8] with a stoichiometry of only 1:1 instead of a homoternary 2:1 complex previously reported for Trp_2_CB[8][9b](Supporting Information, Figures S15 c and S16 c).

Subsequently, the binding behavior of azachalcone to the ternary catalyst complex was investigated. Both ^1^H NMR and fluorescence measurements suggested the inclusion of each of the amino acids and **1 a** in the presence of Cu^2+^, yielding CB[8]⋅**d**⋅Cu^2+^⋅**1 a** and CB[8]⋅**e**⋅Cu^2+^⋅**1 a**. The analysis of the aromatic region of the ^1^H NMR spectra revealed that the signals from both the amino acids and **1 a** were shifted upfield when CB[8] and Cu^2+^ were both present (Supporting Information, Figures S7–S10). Furthermore, the emission of both **d** and **e** were blue-shifted. However, as azachalcone **1 a** was present, quenching was also observed in the study of the full catalytic system (Supporting Information, Figure S12 a, b). These data suggest that the amino acids and **1 a** are indeed in close proximity and included in the CB[8] cavity.

We further explored the role of copper in the complex formation by UV/Vis spectroscopy (Figure 3). Upon binding to the amino acids, the absorption maximum of the dienophile (326 nm) displayed a red-shift (375 nm), while the amino acid absorbance at 275 nm exhibited a fine structure and increased in value (Figure 3 a,b). Notably, titration of CB[8]⋅**d**⋅Cu^2+^ or CB[8]⋅**e**⋅Cu^2+^ into the substrate resulted in a large dienophile absorbance at 375 nm, while the absorbance at 326 nm was drastically decreased (Figure 3 c,d). Moreover, the amino acid absorbance was also red-shifted and the entire spectra generally broadened. These data indicated that the presence of CB[8] dramatically increases the extent of Cu^2+^ bound to **1 a** in solution and suggested the formation of a strong charge-transfer adduct.

**Figure 3:**
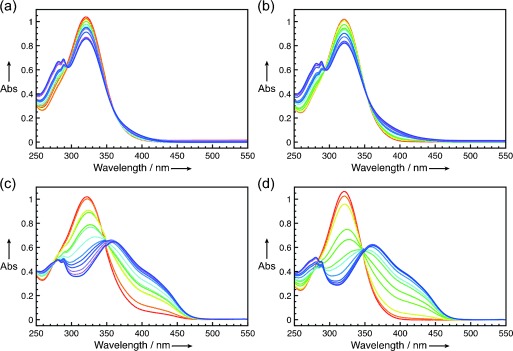
Titration of a) d⋅Cu^2+^, b) e⋅Cu^2+^, c) CB[8]⋅d⋅Cu^2+^, and d) CB[8]⋅e⋅Cu^2+^ to azachalcone 1 a (50 μmol in 1 mm PBS buffer) followed by UV/Vis spectroscopy.

Based on the qualitative data obtained through spectroscopic techniques, we further evaluated the interaction of CB[8]⋅**d**⋅Cu^2+^ and CB[8]⋅**e**⋅Cu^2+^ with **1 a** through ITC. The titration of a mixture of amino acid and Cu^2+^ into the dienophile solution resulted in a low-affinity interaction (Supporting Information, Figures S15 a and S16 a), while the titration of CB[8]⋅**d**⋅Cu^2+^ and CB[8]⋅**e**⋅Cu^2+^ into **1 a** showed a strong binding event for both systems (Supporting Information, Figure S17). Although the *K*_eq_ values obtained from the ITC analyses were one order of magnitude higher than those measured by UV/Vis (Table 2), the trend was exactly the same (Supporting Information, Figures S15, S16).

All of the spectroscopic and calorimetric analyses indicated the occurrence of a binding event between both the nanoreactor systems and the substrate **1 a**. The UV/Vis data also suggested the occurrence of a strong charge-transfer interaction between the substrate and the aromatic amino acid within the cucurbit[8]uril cavity, as predicted by the computational calculations and corroborated by the binding constants measured by ITC. Therefore, as a consequence, one of the two faces on which the diene can approach the dienophile is more shielded, which is, again, in agreement with the results obtained by the computational study and explains the enhanced enantioselectivity achieved in the presence of CB[8].

Taking the best performing combination CB[8]⋅**e**⋅Cu^2+^, the scope of the reaction was investigated using other chalcones **1 b**–**d** under the optimized conditions (Figure 4). Different behaviors of the catalytic system were observed with the varying substrates. Introducing a substituent on the phenyl ring (**1 c** and **1 d**) had little influence on the reaction, meaning that the presence of CB[8] enhanced the enantioselectivity by around 20 % *ee*. However, when a substituent is introduced on the pyridine ring as in **1 b**, the presence of CB[8] did not result in an obviously increased selectivity. To clarify the possible reasons for this finding, we performed the same spectroscopy studies as described before for substrate without substitution on the aromatic heterocycle (Supporting Information, Figure S13). Unlike **1 a**, in the presence of CB[8], the absorption of **1 b** remained unchanged. Furthermore, the distinct red-shift was not observed. These results suggest that **1 b** is most likely not included inside the cavity of CB[8], which can be explained by the steric hindrance from the additional methyl group in close proximity to the metal center. Therefore, these results not only support that pre-inclusion of the substrate is necessary to achieve enantiomeric enhancement, but also suggest that the pyridine ring is included in the cavity during catalysis, while the phenyl ring of the substrate is protruding out of the cavity. These experimental data are also in full agreement with the computational studies performed.

**Figure 4:**
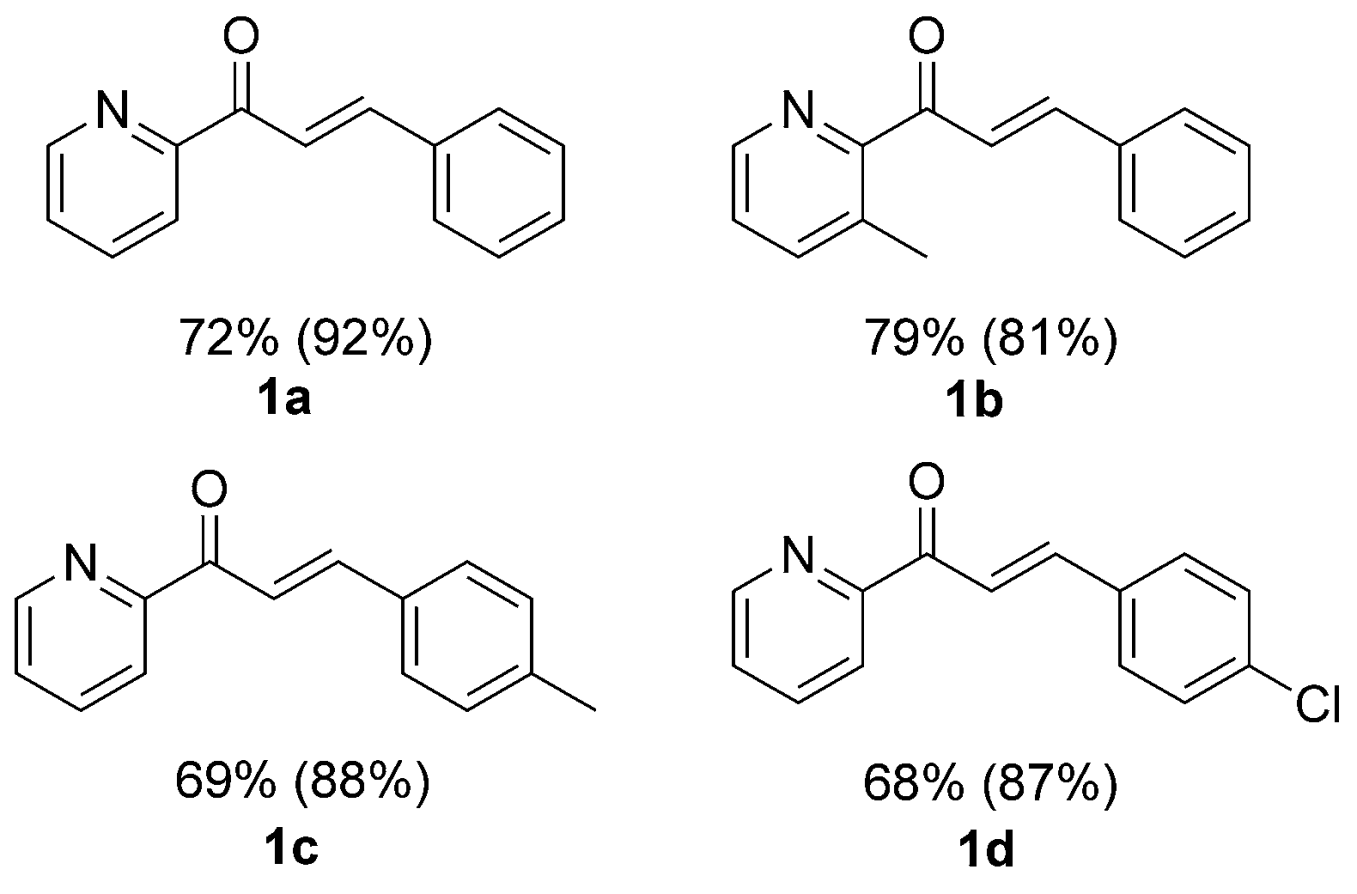
Different substrates employed for the D-A reaction catalyzed by d⋅Cu^2+^ and CB[8]⋅e⋅Cu^2+^. Yields are given (values for CB[8]⋅e⋅Cu^2+^ are shown in brackets).

In conclusion, we have demonstrated the first example of employing achiral cucurbit[8]uril as a nanoreactor in asymmetric catalysis. Using a single amino acid as the chiral source, Cu^2+^ as the Lewis acid, and CB[8] as the host, we obtain a complex that is able to catalyze D-A reactions with high enantioselectivities of up to 92 % *ee*, which are comparable to other bio-inspired catalytic systems containing much larger DNA or protein scaffolds.[15, 19] The high selectivities are also accompanied by a large 9.5-fold rate acceleration of the reaction. With the help of computational studies, ^1^H NMR, optical spectroscopic measurements, and ITC, information about the most likely catalytic mechanism was discerned. During catalysis, the aromatic side chain of the amino acid and the pyridine ring of the dienophile both complexed to copper are located inside the cavity of CB[8], while the vinylbenzene unit is protruding towards the solvent. This spatial arrangement is not only responsible for face-preferential attack of the diene but also for the rate acceleration, since ordered water molecules are regained by the bulk from the dienophile as well as from the hydrophobic CB[8] cavity. The design of an asymmetric supramolecular catalytic system containing a chiral transition-metal complex and a non-chiral macrocyclic pocket is described herein, which is distinct from the traditional concept of introducing an achiral catalyst into an existing chiral scaffold.[20] An obvious advantage of the current approach is the dramatically decreased quantity of expensive chiral resources. As such, we believe that this work will pave the way towards more types of asymmetric reactions involving achiral cucurbit[*n*]uril and other synthetic hosts exhibiting strong binding of auxiliary chiral guests.

## Supporting Information

As a service to our authors and readers, this journal provides supporting information supplied by the authors. Such materials are peer reviewed and may be re-organized for online delivery, but are not copy-edited or typeset. Technical support issues arising from supporting information (other than missing files) should be addressed to the authors.

miscellaneous_information
