# Supplementary material for: Turning Cucurbit[8]uril into a Supramolecular Nanoreactor for Asymmetric Catalysis
Source: Angew Chem Int Ed Engl. 2015 Sep 7;54(44):13007–11. doi: 10.1002/anie.201505628 (PMC4643185; doi:10.1002/anie.201505628)
Supplement: Supplementary file 1 — miscellaneous_information [file anie0054-13007-sd1.pdf]

Angewandte Chemie
Eine Zeitschrift der Gesellschaft Deutscher Chemiker

Supporting Information

Turning Cucurbit[8]uril into a Supramolecular Nanoreactor for Asymmetric Catalysis

Lifei Zheng, Silvia Sonzini, Masyitha Ambarwati, Edina Rosta, Oren A. Scherman, and Andreas Herrmann**

anie_201505628_sm_miscellaneous_information.pdf

1. General

All UV-Vis spectra were measured either on a JASCO V-630 or a Cary 400 spectrophotometer, both equipped with a temperature control accessory. All fluorescence measurements were recorded on a Cary Eclipse fluorimeter equipped with a temperature control accessory. Nuclear magnetic resonance (NMR) spectroscopy was carried out either on a Varian 400 (^{1}H, 400 MHz), to characterize small molecules, or on a Bruker Advance 500 (^{1}H, 500 MHz), to characterize the nanoreactors. Chemical shift (δ) are quoted in ppm with the internal reference tetramethylsilane. Isothermal calorimetry (ITC) measurements were carried out at 25°C with a MicroCal Auto-ITC. Enantiomeric excess was determined by high-performance liquid chromatography (HPLC) analysis performed on a Shimadzu 10AD-VP instrument with the eluents of heptane and isopropanol (*i*-PrOH), using a Daicel Chiralcel ODH column, OD or AD column. Conversions were determined by HPLC and corrected for differences in extinction coefficient as reported in the literature.[1]

2. Materials

CB[n] was synthesized from glycoluril with formaldehyde by a basic procedures published by Day and Kim, further isolation and purification were carried out by our previously reported method.[2] Substrates **1a-d** were prepared following published procedures.[3] All other chemicals were acquired from Acros or Sigma Aldrich and were used without further purification. For all experiments, ultrapure water (18.2 MΩ) purified by a MilliQ-Millipore system (Millipore, Germany) was used.

3. Optimization of the D-A reaction

Taking the identified assembly CB[8]•**d**•Cu^{2+} as an example, we investigated the effects of different reaction conditions on selectivities. As a first important parameter, the stoichiometry between the ligands and copper ion was investigated (Table 2, entries 1-5). A CB[8]/**d** /Cu^{2+} ratio of 2/1.5/1 gave the best *ee* value; while increasing the amount of either amino acid or Cu^{2+} induced a progressive reduction in the *ee* values approaching 45% for CB[8]/**d**/Cu^{2+}= 1/2/2. In addition, various buffer conditions with different pH values were investigated (entries 6-8), maximum *ee* values were obtained at a pH of 7.4. However, at pH 9, the *ee* value dropped slightly, while decreasing the pH to 5.5 resulted in a significant decrease in the

enantioselectivity (40% *ee*). Finally, a decrease in the catalyst loading from 3 to 0.5 mol% led to only a slight drop of the conversion and *ee* value (entry 9). Hence, we retain high enantioselectivity at extremely low catalyst loading, which is especially important in the case of CB[8] with its limited solubility in water.

Table S1. Results of Diels-Alder reactions based on CB[8]•d•Cu^{2+} under various buffer conditions

Entry	Ratio of CB [8]/**d** / Cu^{2+}	Buffer[a]	*ee*(endo) [%][b]
1	1 / 1 / 1	MOPS (pH 6.5)	57
2	2 / 1.5 / 1	MOPS (pH 6.5)	67
3	3 / 1.5 / 1	MOPS (pH 6.5)	65
4	1 / 2 / 1	MOPS (pH 6.5)	60
5	1 / 2 / 2	MOPS (pH 6.5)	45
6	2 / 1.5 / 1	MES (pH 5.5)	40
7	2 / 1.5 / 1	PBS (pH 7.4)	73
8	2 / 1.5 / 1	PBS (pH 9.0)	65
9[c]	2 / 1.5 / 1	PBS (pH 7.4)	72

[a] MOPS denotes 3-(N-morpholine) propanesulfonic acid buffer; MES denotes 2-(N-morpholine)ethanesulfonic acid buffer. [b] Data were obtained from the crude product on chiral HPLC. The *ee* values are averaged over two experiments and are reproducible within ±2%. [c] Cu^{2+} (0.5mol %), **a-e** (0.75 mol %), CB [8] (1 mol %) were used.

4. Kinetic and thermodynamic measurements

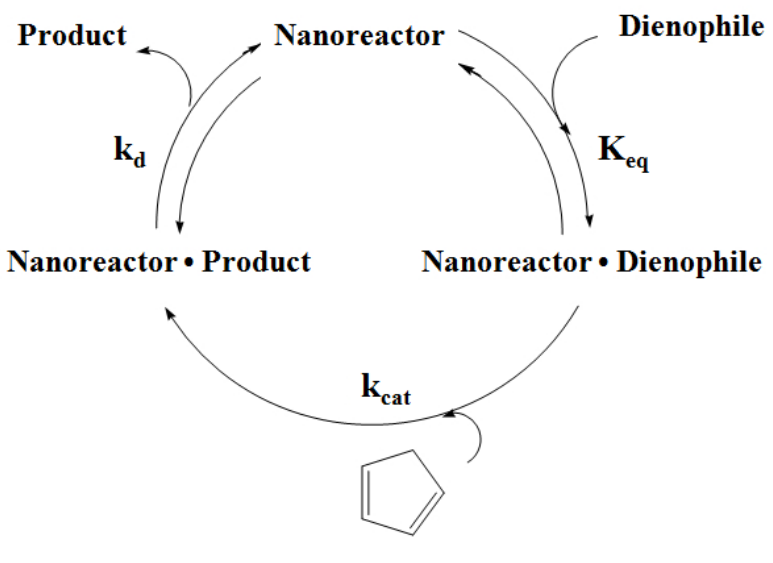

Scheme S1. Proposed catalytic cycle of D-A reaction.

All kinetic and thermodynamic measurements were performed as reported by Otto and Engberts.[3] A typical procedure of measuring k_{app} is as follows: the samples contained 112 μM CB[8], 84 μM amino acid, 56 μM $Cu(NO_3)_2$, 22 μM azachalcone (**1a**), and 3.67 mM (2.5 mM in the case of CB [8]•**e**•Cu^{2+}) of cyclopentadiene (**2**) with a total volume of around 2.1 mL in 1mM PBS buffer at pH=7.4. The apparent second-

order rate constant (k_{app}) was determined according to the following expression:

$$k_{app} = d[A_{\mathbf{1a}}]/dt \cdot (d \cdot (\varepsilon_{\mathbf{1a}} - \varepsilon_{\mathbf{3a}}) \cdot [\mathbf{1a}]_0 \cdot [\mathbf{2}]_0)^{-1}$$

In which $d[A_{\mathbf{1a}}]/dt$ is the slope of the absorption of **1a** at a certain wavelength *vs*. time for the first 10% decrease of substrate conversion. d is the path length of the cuvette and $\varepsilon_{\mathbf{1a}}$and $\varepsilon_{\mathbf{3a}}$represent the extinction coefficients of **1a** and **3a**, respectively.

4.1 Determination of the molar extinction coefficients:

Since the maximum absorption wavelength of **1a** was significantly influenced by the presence of CB[8], the molar extinction coefficient of **1a** and **3a** were determined at various wavelengths which were related to the different catalyst combinations. The absorbance of **1a** and **3a** at a certain wavelength was measured with the fixed concentration of 10, 20, 30, 40, and 50 μM. The molar extinction coefficient was estimated from the slope of the fitting curve.

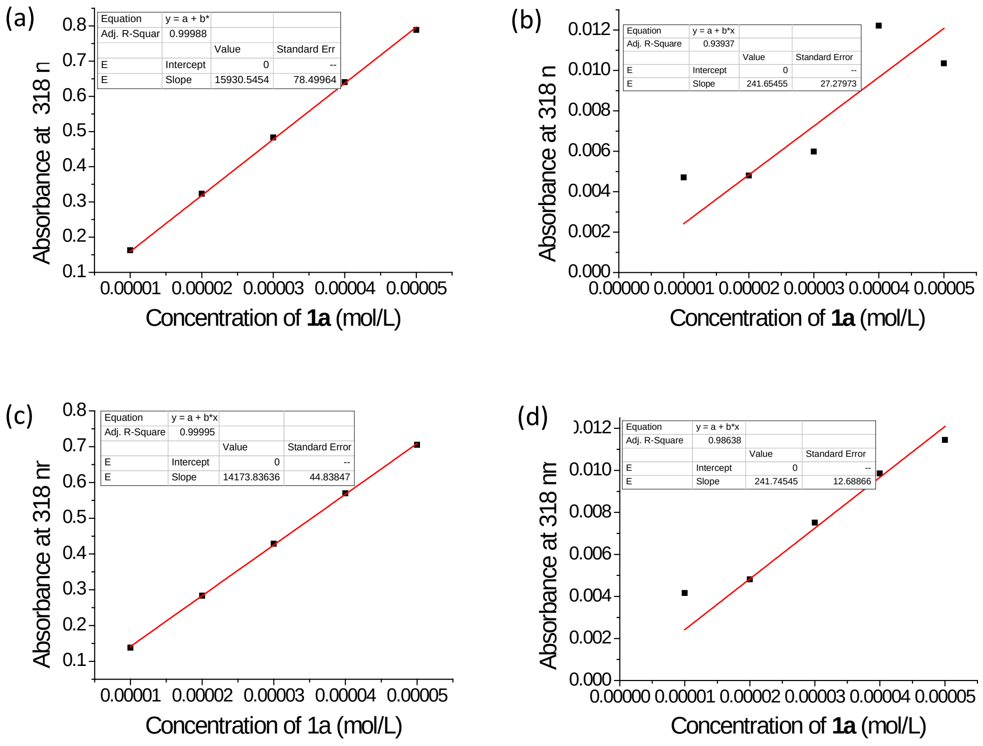

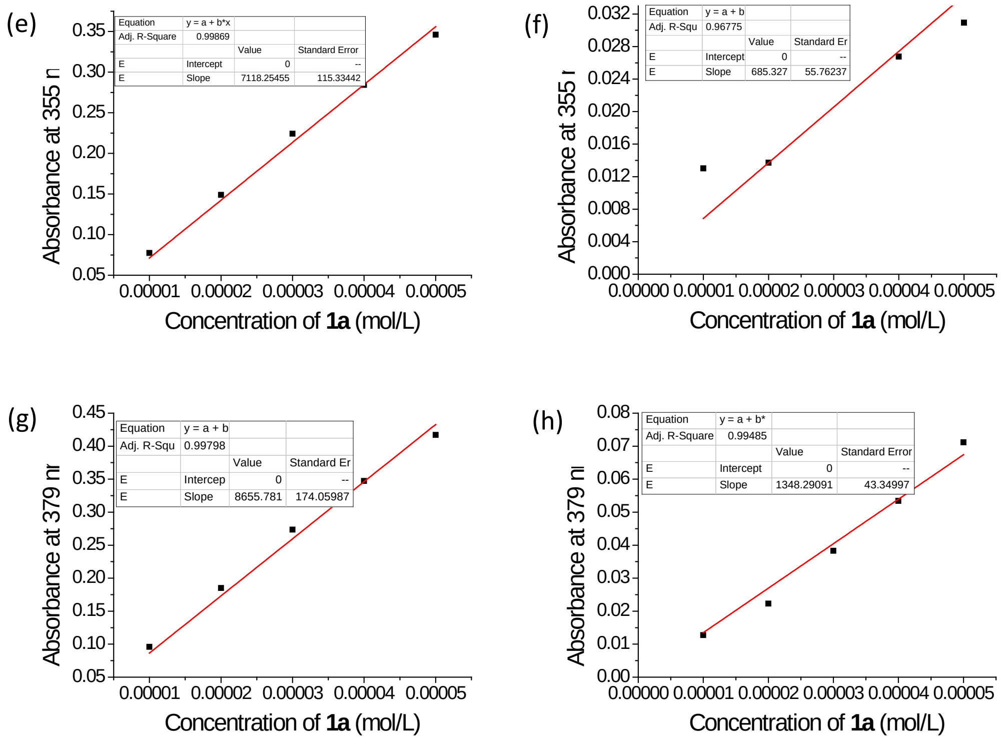

Figure S1 The molar extinction coefficient of **1a** and **3a** in the presence of **d**•Cu^{2+} (a and b); **e**•Cu^{2+} (c and d); CB [8]•**d**•Cu^{2+} (e and f); CB [8]•**e**•Cu^{2+} (g and h).

4.2 Determination of the equilibrium constants K_{eq}:

At 25°C, equilibrium constants were obtained by measuring the extinction coefficient of partially complexed dienophile (ε_{obs}) at a proper wavelength where the extinction coefficient of uncomplexed and complexed **1a** show maximal differences. ε_{obs}as a function of the concentration of copper ion can be derived as following:

$$[Cu^{2+}]/(\varepsilon_{1a} - \varepsilon_{obs}) = 1/(\varepsilon_{1a}–\varepsilon_{complex})\cdot K_{eq} + [Cu^{2+}]/(\varepsilon_{1a}–\varepsilon_{complex})$$

ε_{1a} and $\varepsilon_{complex}$ represent the extinction coefficients of the uncomplexed and complexed dienophile, respectively. When $[Cu^{2+}]/(\varepsilon_{1a} - \varepsilon_{obs})$ was plotted *vs.*$[Cu^{2+}]$, a linear relation was observed, which was used to determine the equilibrium constant K_{eq} which equals the ratio of slope and intercept.

The concentration of copper complex was varied from 0.06-0.6 mM in the absence of CB[8], and 0.03-0.15 mM in the presence of CB[8], while the typical concentration range of dienophile was 6-20 μM. Under each condition, the K_{eq} was measured from two independent experiments.

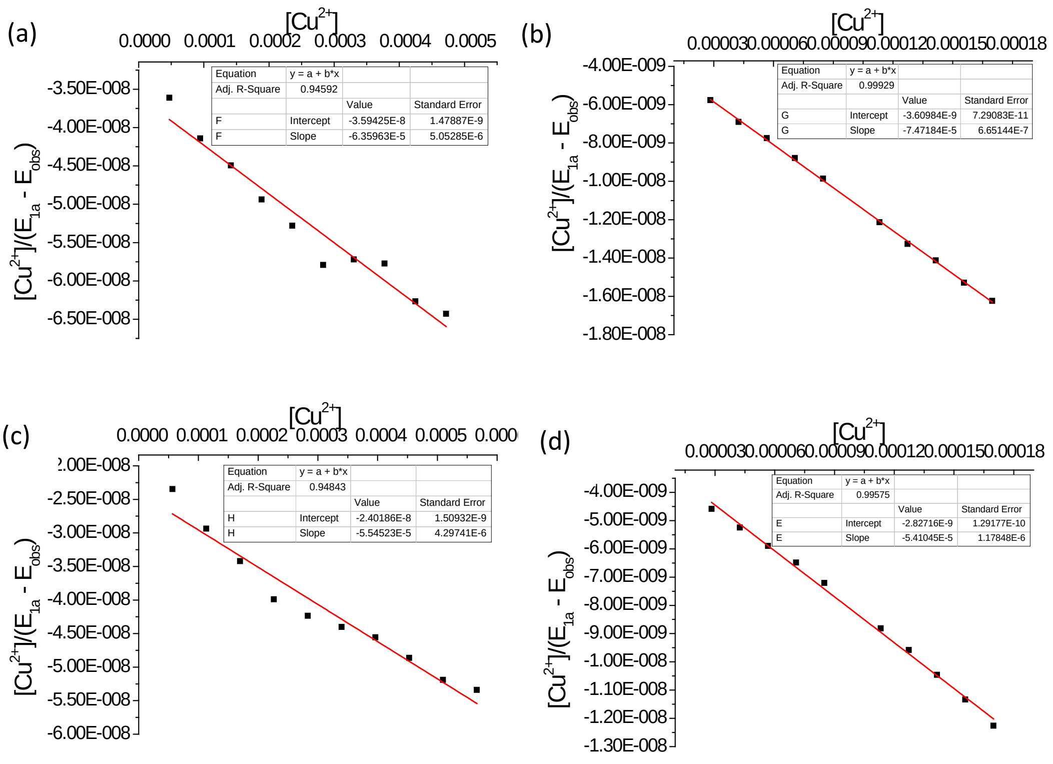

Figure S2. The plot of $[Cu^{2+}]/(\varepsilon_{1a} - \varepsilon_{obs})$ *vs.*$[Cu^{2+}]$: (a) **d**•Cu^{2+}; (b) CB [8]•**d**•Cu^{2+}; (c) **e**•Cu^{2+}; and (d) CB [8]•**e**•Cu^{2+}.

4.3 Determination of k_{cat}:

The k_{cat} values were calculated according to the following equation:

$$k_{app} = \frac{k_0 * [\mathbf{1a}]_f}{[\mathbf{1a}]_t} + \frac{K_{eq} * [Cu^{2+}]_f}{K_{eq} * [Cu^{2+}]_f + 1} * k_{cat}$$

k_0 is the rate constant of D-A reaction in the absence of any catalyst. $[\mathbf{1a}]_f$ is the concentration of free **1a**, $[\mathbf{1a}]_t$ is the concentration of total amount of **1a**; $[Cu^{2+}]_f$ is the concentration of free copper complex. Since $k_0 \ll k_{cat}$, the contribution of the uncatalyzed reaction can be neglected and this equation simplifies to:

$$k_{app} = \frac{K_{eq} * [Cu^{2+}]_f}{K_{eq} * [Cu^{2+}]_f + 1} * k_{cat}$$

Herein, the concentration of unbound copper complex can be calculated from the measured K_{eq}.

4.4 Eyring plot of k_{app} at temperatures ranging from 288 to 308 K:

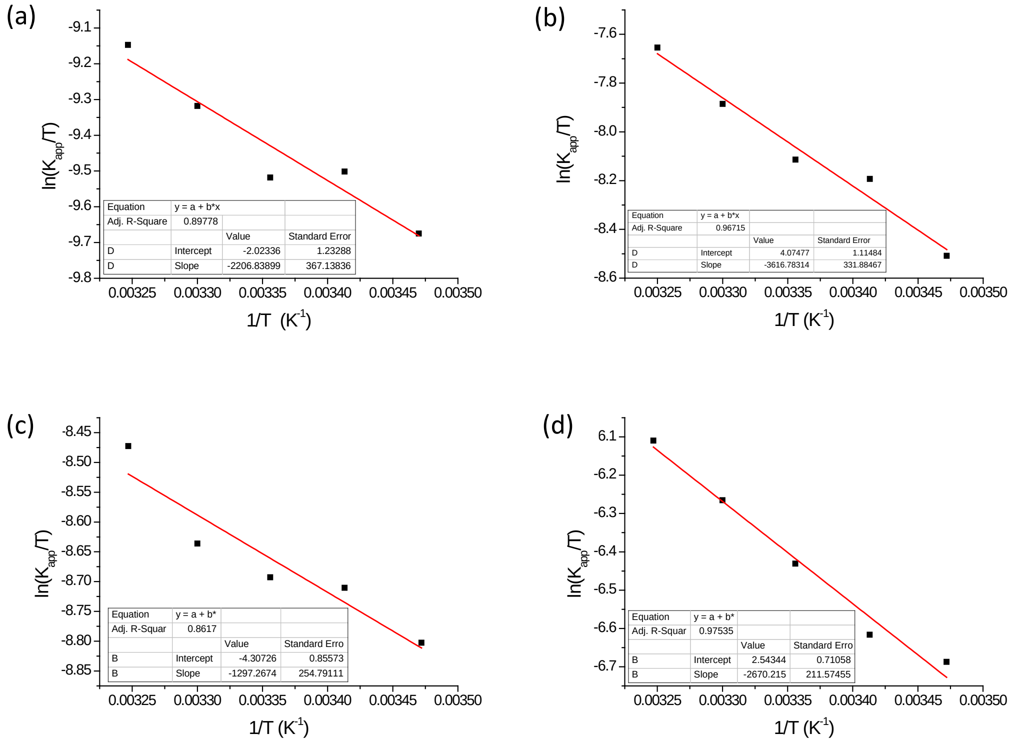

Figure S3. Eyring plot of the apparent rate constant of D-A reactions catalyzed by**d**•Cu^{2+} (a); CB [8]•**d**•Cu^{2+} (b); **e**•Cu^{2+} (c); and CB [8]•**e**•Cu^{2+} (d).

5. Computational studies

The geometries of the catalytic complexes both with and without CB[8] were optimized using DFT B3LYP with Grimme D3 dispersion correction[4] and 6-31G* basis set.Additionally, a polarizable continuum solvent model (PCM)[5]was used throughout the calculations to represent the standard water dielectric medium.

5.1 Geometric isomers of azachalcone in complex with Cu^{2+} and Trp

Optimized geometries for isomers of the complex tryptophan-Cu^{2+}-azachalcone were calculated using DFT B3LYP/6-31G* level of theory with Grimme D3 dispersion corrections[4] and the PCM continuum solvent model as implemented in Gaussian09[5]. Additional calculations were also run with a single water molecule explicitly,to take into account possible solvent interactions with the copper ion. Subsequently, single point energies were determined with the aug-ccpVTZ-PP basis set at the same level of theory. The trans-cisoid conformation was found as the most stable isomer.

**Azachalcone - Cu^{2+} - tryptophan**	**ΔE[a](kcal/mol)**	**ΔE with single explicit water molecule (kcal/mol)**	
		6-31G*	aug-ccpVTZ-PP
trans - cisoid	**0**	**0**[b]	**0**
cis - cisoid	5.0	4.7	4.8
trans-transoid	5.6	5.4	5.2
cis - transoid	9.7	-	-

Table S2. Relative energies calculated for different isomers of the complex in the pi-stacking conformation. The reference system presenting the lowest energy is set at 0 kcal/mol.

[a]These relative energies were determined with aug-ccpVTZ-PP basis set.

[b]This structure is represented in Figure S4a.

Once the complex was inserted in CB[8], in the presence of a single explicit water molecule, the energy was minimized again to assure that the copper is indeed coordinated to the water molecule and not to the macrocyle rim(see Figure S4b).

Therefore, optimized geometries for the complex with and without CB[8] in presence of cyclopentadiene (Cp) approaching from different directions were alsocalculated as described above. Since the Cp approach was significantly hindered from the Trp-side attack and the geometry optimizations resulted in a non-reactive conformation, a

distance constraint was added in all the calculations to obtain models closely related to the transition state. In all of the approaches studied, two constraints were set at 3.0 Å each between the two pairs of reacting carbon atoms. Oncewe obtained the optimized geometries, the constraints were released and the unconstrained optimum geometries calculated as well. Note that only the 6-31G* basis set was used in the calculations with CB[8] on account of the large number of atoms to analyze when the macrocycle is included. As a general trend, we observed similar but slightly lower energy differences between the various approach conformations when using the larger basis set (aug-ccpVTZ-PP), both in the calculations of the complex on its own and in presence of Cp (Tables S1 and S2). Therefore,it is reasonable to assume that the relative energies obtained for the complex with CB[8] would have only been lower if calculated with the larger basis set. The obtained relative energies (Table S2) suggested that the endo complex is slightly favored as compared to the exo complex. The Trp-side approach was much less favorable both with and without CB[8], in addition, the complex seemed totwist and open slightly to allow the Cp to interact with the azachalcone (Figure 2b and S6). Importantly, the energies obtained are in good agreement with the product ratios obtained experimentally.

**Relative energies (kcal/mol)**	**Cp approach opposite side Trp**		**Cp approach same side Trp**	
	Endo	Exo	Endo	Exo
Azachalcone - Cu^{2+} - Trp (6-31G* basis set)	**0**	1.0	4.4	7.5
Azachalcone - Cu^{2+} - Trp (aug-ccpVTZ-PP basis set)	**0**	0.9	3.1	6.4
Azachalcone - Cu^{2+} - Trp - CB[8] (6-31G* basis set)	**0**	0.2	6.5	10

Table S3. Relative energies calculated for different approaching directions of Cp towards the complex with or without CB[8]. The reference system presenting the lowest energy is set at 0 kcal/mol.

5.2 Geometry optimizations

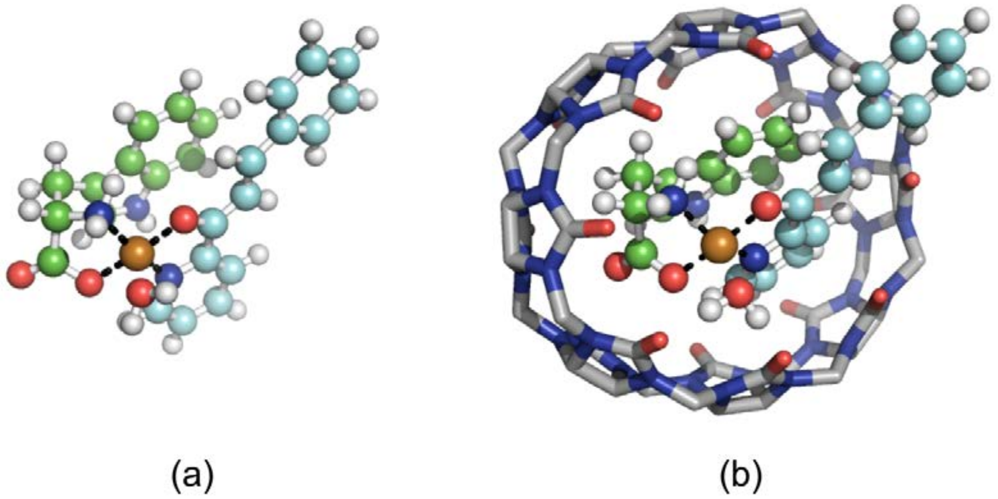

Figure S4. Azachalcone-Cu^{2+}-Trp complex in the trans-cisoid conformation, the most stable, (a) by itself and (b) inside CB[8] cavity.

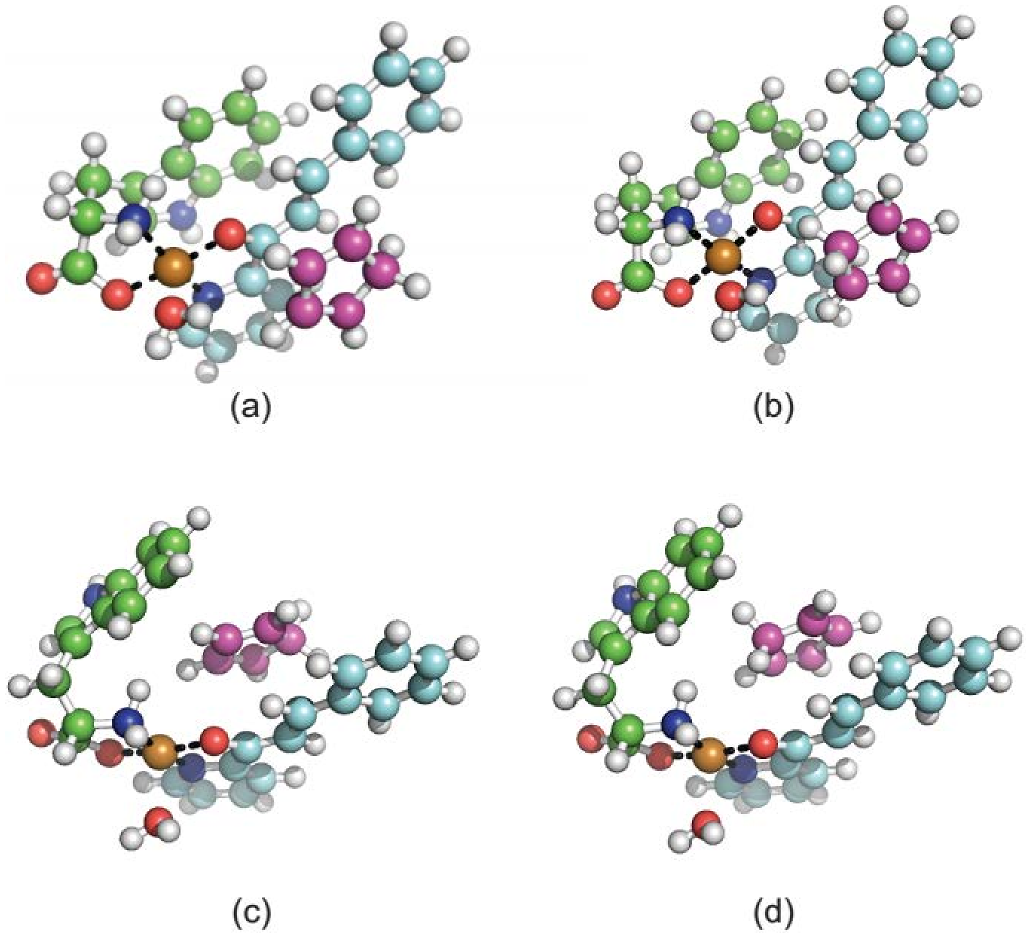

Figure S5. Azachalcone-Cu^{2+}-Trp complex in the trans-cisoid conformation with Cp approaching: from the opposite side of Trp in the (a) endo and (b) exo direction; from the same side of Trp in the (c) endo and (d) exo direction.

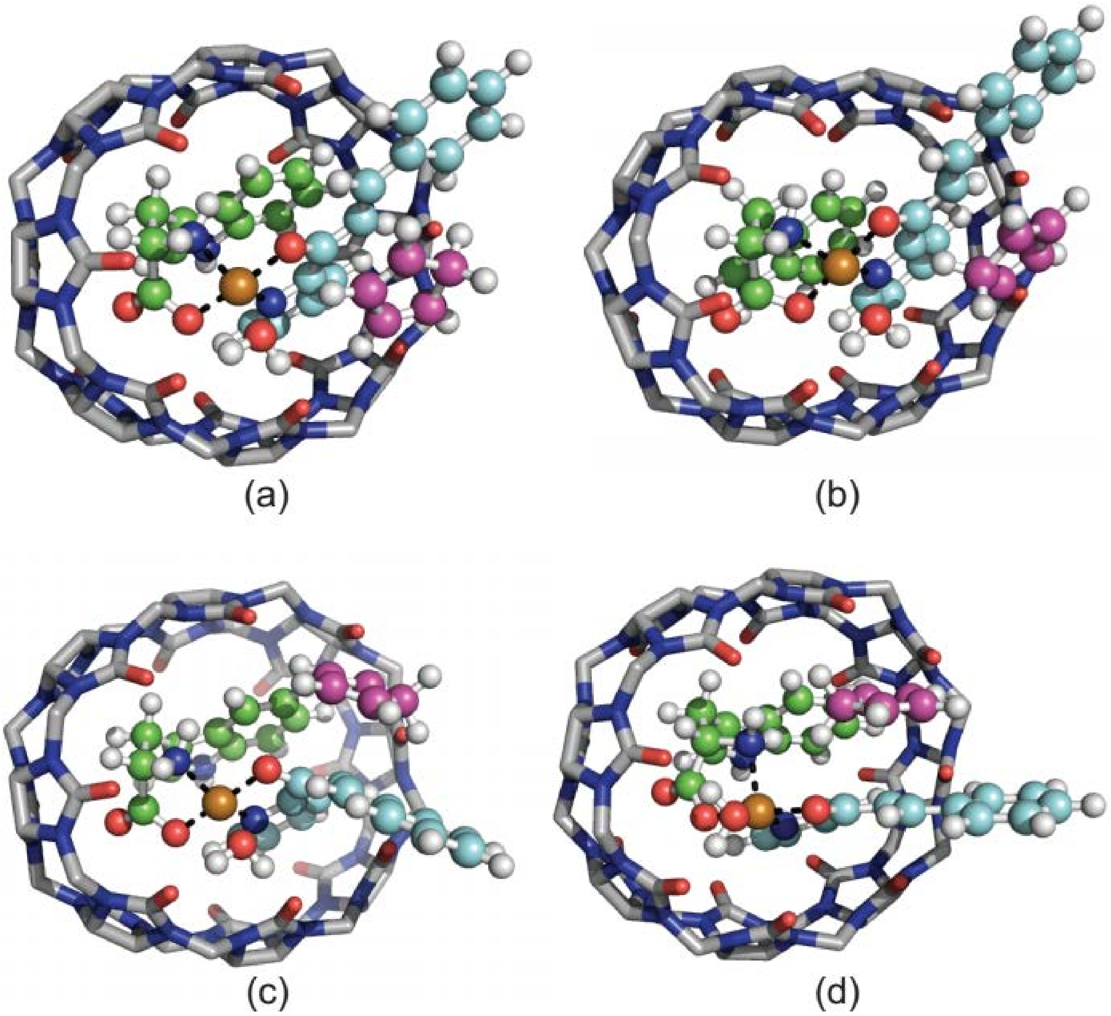

Figure S6. Azachalcone-Cu^{2+}-Trp-CB[8] complex in the trans-cisoid conformation with Cp approaching: from the opposite side of Trp in the (a) endo and (b) exo direction; from the same side of Trp in the (c) endo and (d) exo direction.

6.^{1}H-NMR

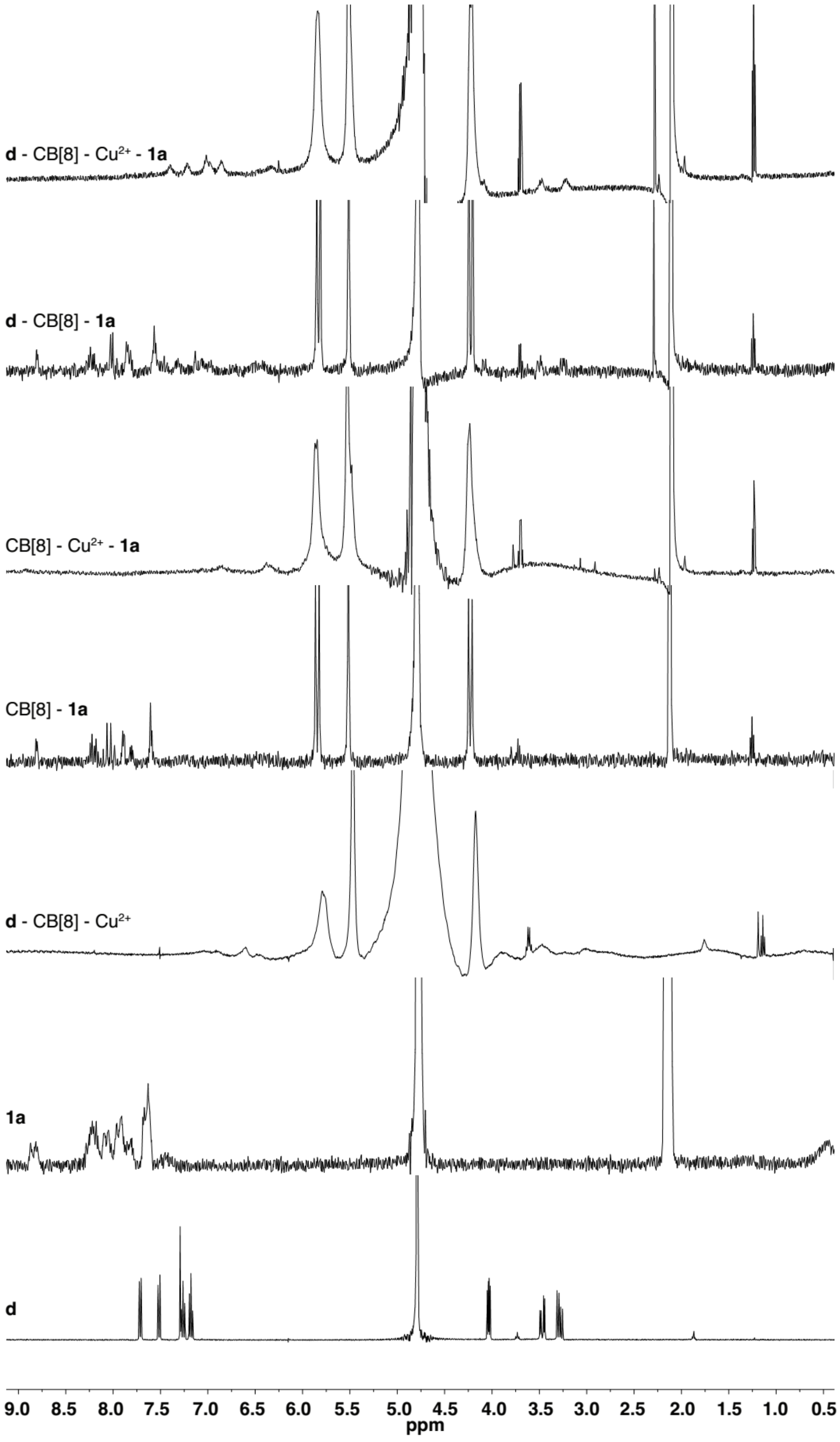

Figure S7. ^{1}H-NMR studies of different **d**, Cu^{2+}, CB[8] and **1a** mixtures. Each component in each sample was dissolved at 0.5 mM in D_2O, for **1a** 10% of D_3-ACN was added to dissolve it.

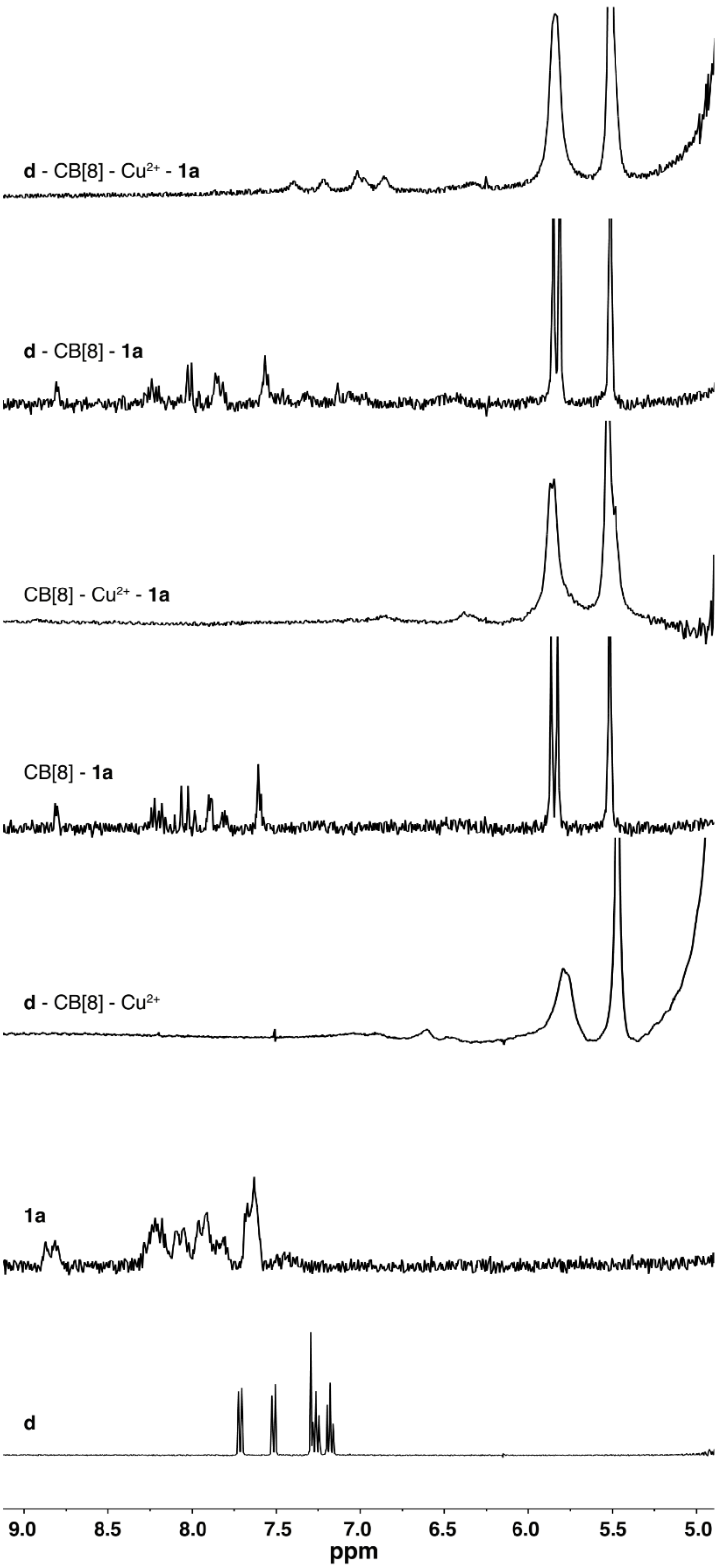

Figure S8. Enlargement of the aromatic region of ^{1}H-NMR studies of different **d**, Cu^{2+}, CB[8] and **1a** mixtures. Each component in each sample was dissolved at 0.5 mM in D_2O, for **1a**10% of D_3-ACN was added to dissolve it.

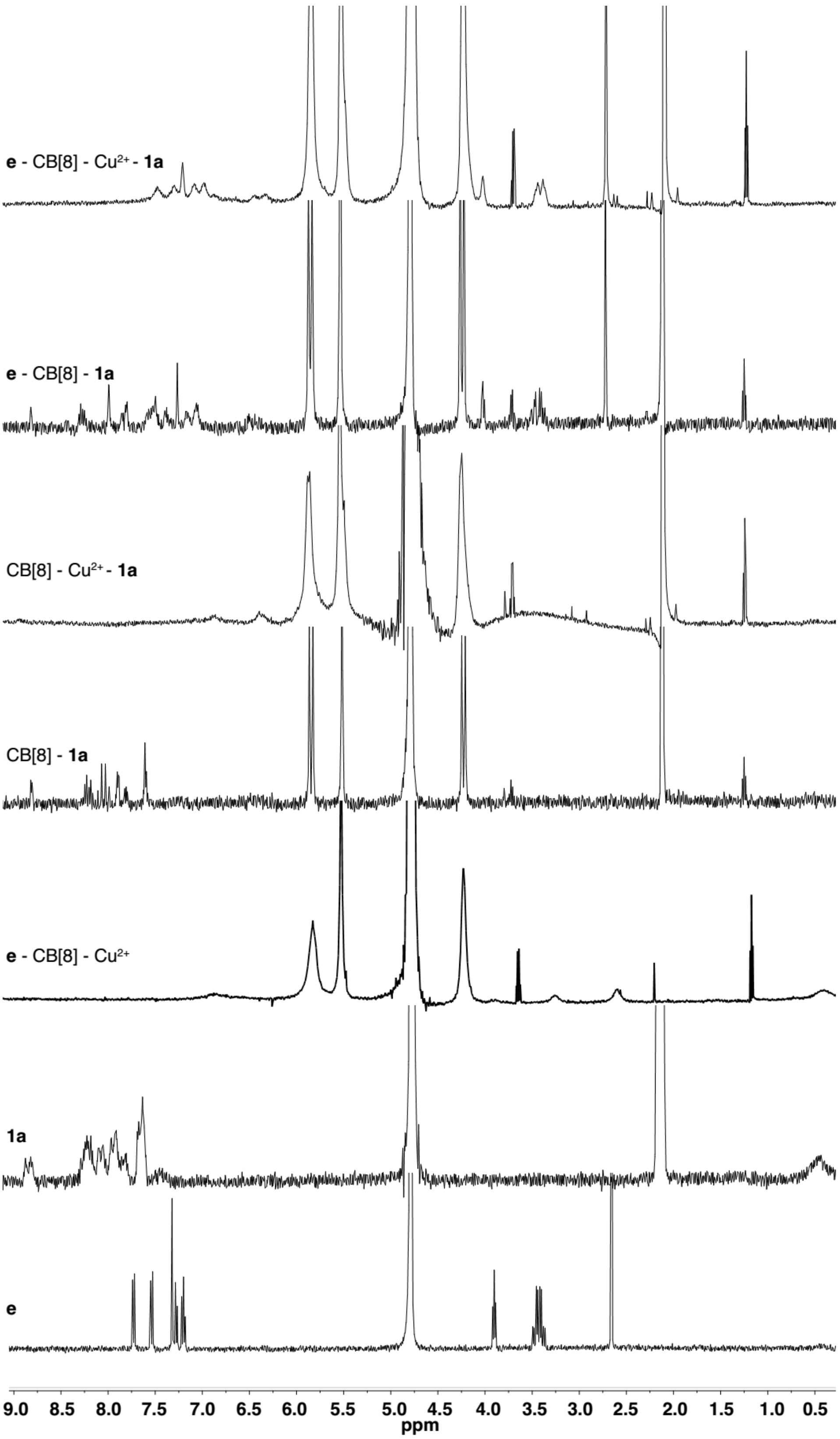

Figure S9. ^{1}H-NMR studies of different **e**,Cu^{2+}, CB[8] and **1a** mixtures. Each component in each sample was dissolved at 0.5 mM in D_2O, for **1a** 10% of D_3-ACN was added to dissolve it.

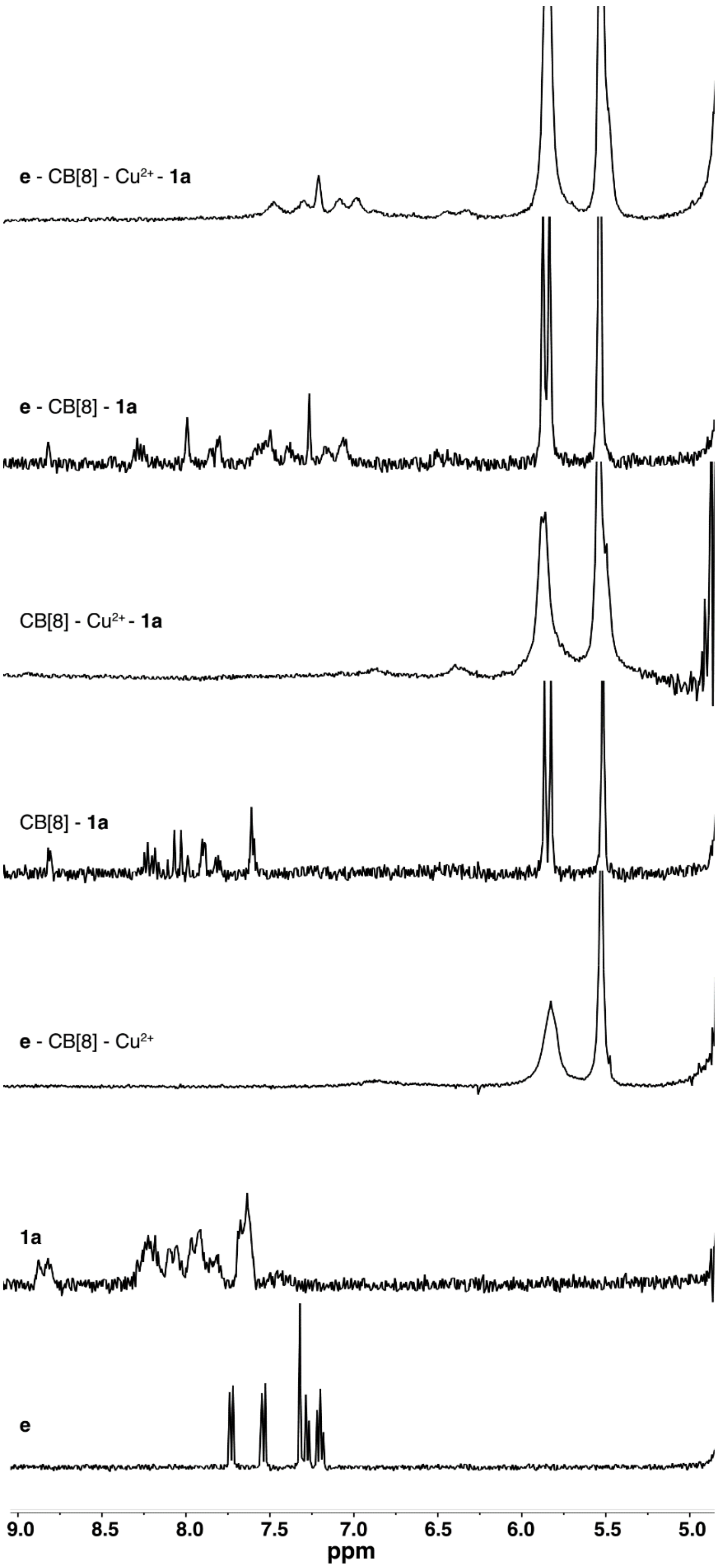

Figure S10. Enlargement of the aromatic region of ^{1}H-NMR studies of different **e**, Cu^{2+}, CB[8] and **1a** mixtures. Each component in each sample was dissolved at 0.5 mM in D_2O, for **1a** 10% of D_3-ACN was added to dissolve it.

7. UV-Vis and fluorescence spectroscopy

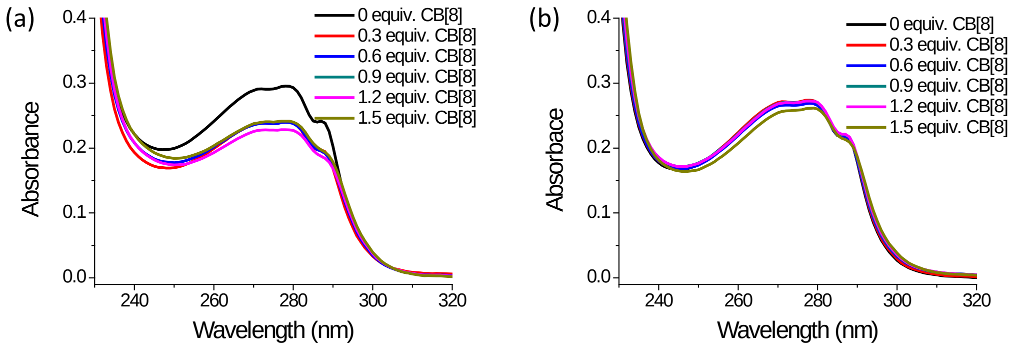

Figure S11. Titration of CB[8] (0-1.5 equiv.) into (a) **d** •Cu^{2+} and (b) **e**•Cu^{2+} both dissolved in 1 mM PBS buffer followed by UV-vis spectroscopy.

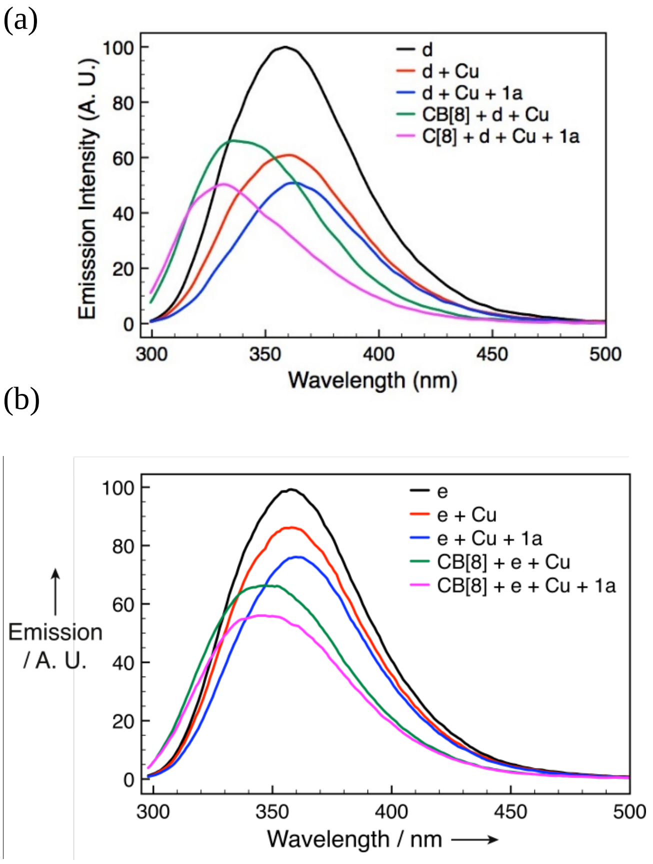

Figure S12. Fluorescence spectra of aromatic amino acids (a) **d** and (b) **e** in presence of different catalyst components and substrate**1a**.

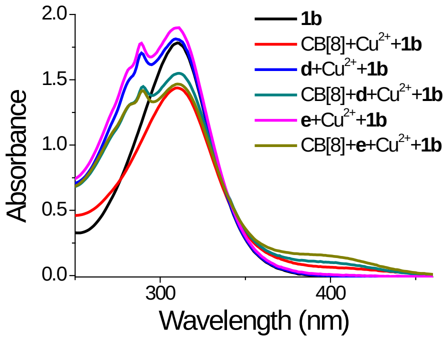

Figure S13. UV-vis spectra of azachalcone**1b** under different conditions.

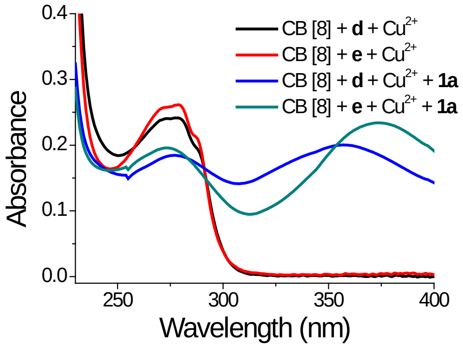

Figure S14. UV-vis spectra of CB[8] supramolecular catalysts before and after addition of azachalcone.

8. Isothermal Calorimetry (ITC) analyses

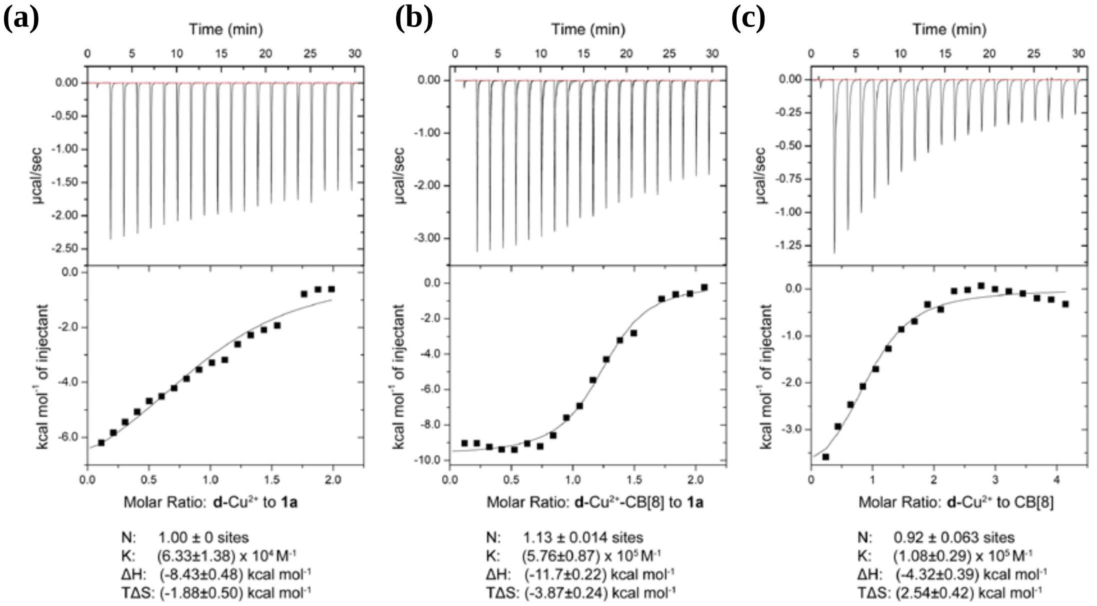

Figure S15. ITC of (a) **d**•Cu^{2+} (1:1 mixture 500 μM in water) and (b) CB[8]•**d**•Cu^{2+} (1:1:1 mixture 500 μM in water) into azachalcone **1a** (50 μM in 2% ACN/water). ITC of (c) **d**•Cu^{2+}(1:1 mixture 1 mM in water) into CB[8] (50μM in water).

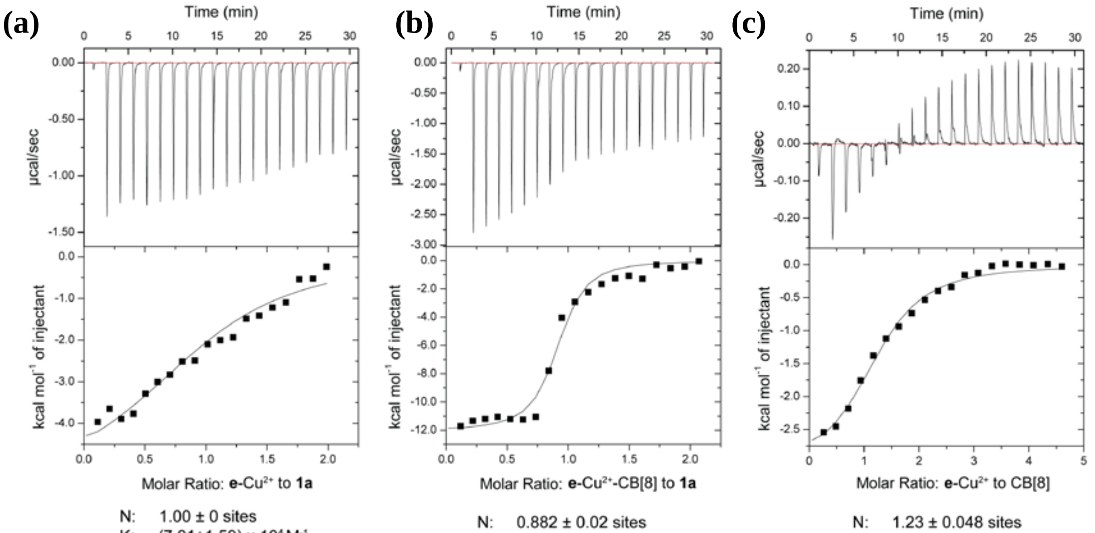

Figure S16. ITC of (a) **e**•Cu^{2+} (1:1 mixture 500 μM in water) and (b) CB[8]•**e**•Cu^{2+} (1:1:1 mixture 500 μM in water) into azachalcone **1a** (50 μM in 2% ACN/water). ITC of (c) **e**•Cu^{2+} (1:1 mixture 1 mM in water) into CB[8] (50 μM in water)

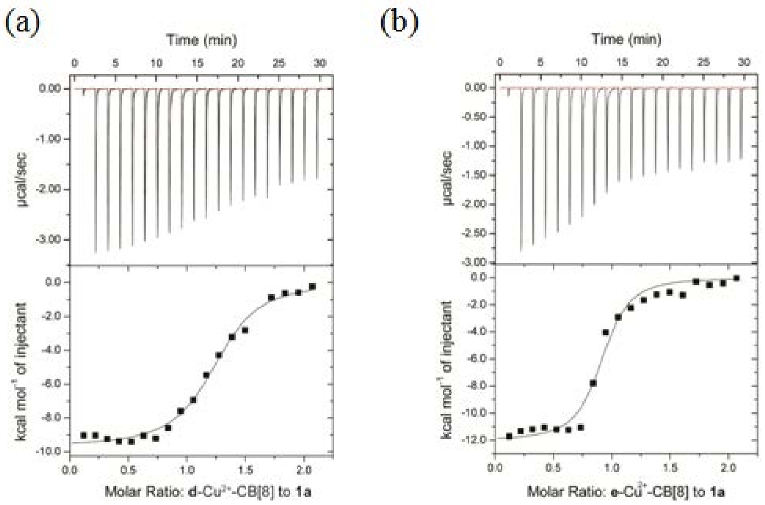

Figure S17. ITC of (a) CB[8]•**d** •Cu^{2+} (1:1:1 mixture 500 μM in water), and (b) CB[8]•**e** •Cu^{2+} (1:1:1 mixture 500 μM in water) into azachalcone**1a** (50 μM in 2% ACN/water).

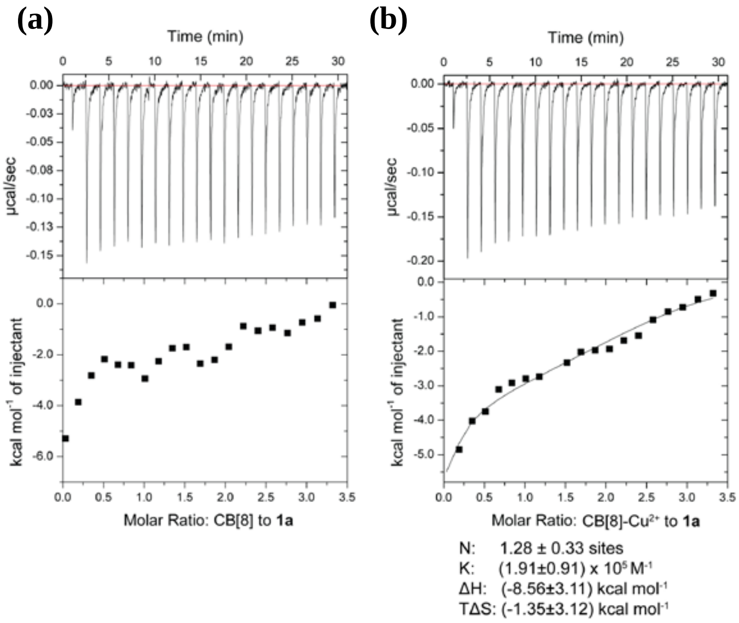

Figure S18 ITC of (a) CB[8] (80μM in water) and (b) CB[8]•Cu^{2+} (1:1 mixture 80 μM in water) into azachalcone **1a** (5 μM in 10% ACN/water).

9. Synthesis

Standard procedure for Diels-Alder reactions employing cucurbituril nanoreactor:

An aqueous solution composed of 75µL of 1mM amino acid, 50µL of 1 mM $Cu(NO_3)_2$ and 50µL of PBS buffer(10mM, pH 7.4) was shaken for 30minutes.To this components a solution of CB[8] (666µL, 150µM in water) was added, and the mixture was shaken at room temperature for another 30 minutes. Then an aliquot of a stock solution of azachalcone in CH_3CN (10 µL of 150 mM solution) was added and the mixture was cooled to 6 °C. The reaction was started by addition of the freshly distilled cyclopentadiene (4 µL, 48 µmol) and proceeded for 24 h at 6 °C, followed by extraction with diethyl ether (3×1 ml). After removal of the solvent, the *ee* of crude products were directly determined by HPLC on a chiral stationary phase.

Preparation of 2-Acetyl-3-methylpyridine

Under an argon atmosphere, *n*-BuLi (7.3 mL, 11.6mmol, 1.6 M inn-hexane) was added to a mixture of 2-bromo-3-methyl-pyridine (1.0 g, 5.8mmol) and anhydrous Et_2O (30 mL) at -78^{0}C. The reaction mixture was stirred for 30 minutes. Then, N,N-dimethylacetamide (2.0 mL, 23.2 mmol) was added, and the mixture was allowed to reach ambient temperature within 1 h. The reaction mixture was quenched by adding saturated NH_4Cl, and was then extracted with Et_2O three times. The combined organic layers were washed with brine, dried, and concentrated. The crude product was purified on silica gel column using *n*-hexane/AcOEt (10:1) to yield the desired product. ^{1}H-NMR ($CDCl_3$, 400 MHz): 2.57 (s, 3H), 2.70 (s, 3H),7.32 (m, 1H), 7.57 (m, 1H), 8.50 (m, 1H).

Preparation of (E)-1-(3-methyl-2-pyridinyl)-3-phenyl-2-propen-1-one (1b)

A solution of 10% NaOH (2.0 ml) was added dropwise to a mixture of 2-acetyl-3-methylpyridine (0.5 g, 3.7 mmol) and benzaldehyde (0.42 g, 4.0 mmol) in methanol (30 ml) with stirring, and was continued for 12h at room temperature. After evaporation of the solvent under reduced pressure, the crude product was purified by flash chromatography (n-hexane/ethyl acetate = 4/1) to yield the desired product. ^{1}H-NMR ($CDCl_3$, 400 MHz): 2.65 (s, 3H), 7.32 (m, 1H), 7.41 (m, 3H), 7.60 (m, 1H), 7.68 (m, 2H), 7.77 (d, *J*=16.01 Hz, 1H), 8.01 (d, *J*=16.09 Hz, 1H), 8.55 (s,1H).

^{1}H NMR of D-A product

(3-methyl-2-pyridinyl)(3-phenylbicyclo[2.2.1]hept-5-en-2-yl)methanone (3b).

^{1}H-NMR ($CDCl_3$, 400 MHz):1.53 (dd, *J*= 1.71 Hz, *J*= 8.48 Hz,1H), 1.94 (d, *J*=8.44 Hz, 1H), 2.46 (s, 3H), 3.08 (s, 1H), 3.35 (d, *J*= 3.81 Hz, 2H),3.44 (s, 1H), 4.55 (m, 1H), 5.94 (m, 1H), 7.34 (m, 6H), 7.58 (d, *J*= 7.75 Hz, 1H), 8.53 (m, 1H).

10. HPLC traces of D-A product 3a

3a formed by **d**•Cu^{2+}

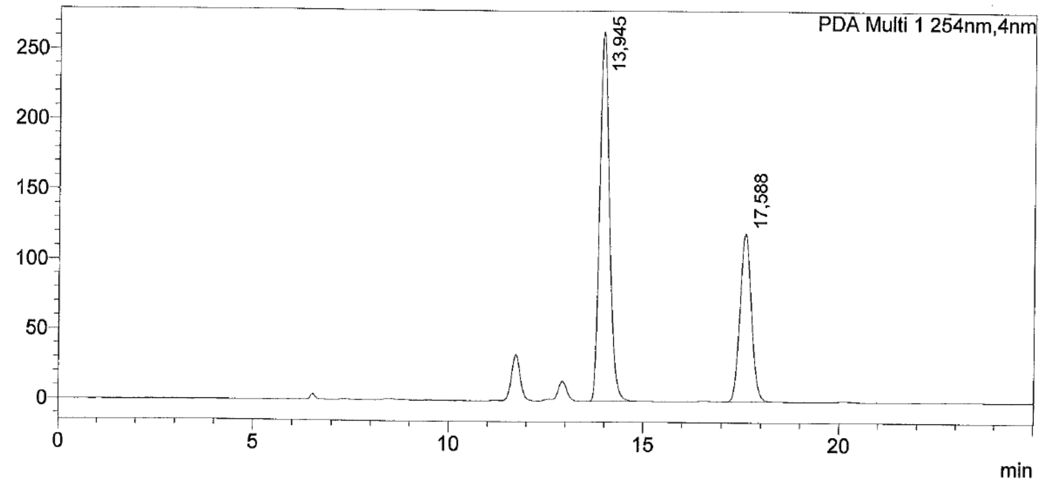

<Peak Table>

Peak Table

AD2

Peak#	Ret. Time	Area	Height	Conc.	Unit	Mark	Name
Total							

PDA Ch1 254nm

Peak#	Ret. Time	Area	Height	Conc.	Unit	Mark	Name
1	13,945	4614111	263769	65,410		M	
2	17,588	2439990	120285	34,590		M	
Total		7054102	384054				

3a formed by CB [8]• **d**•Cu^{2+}

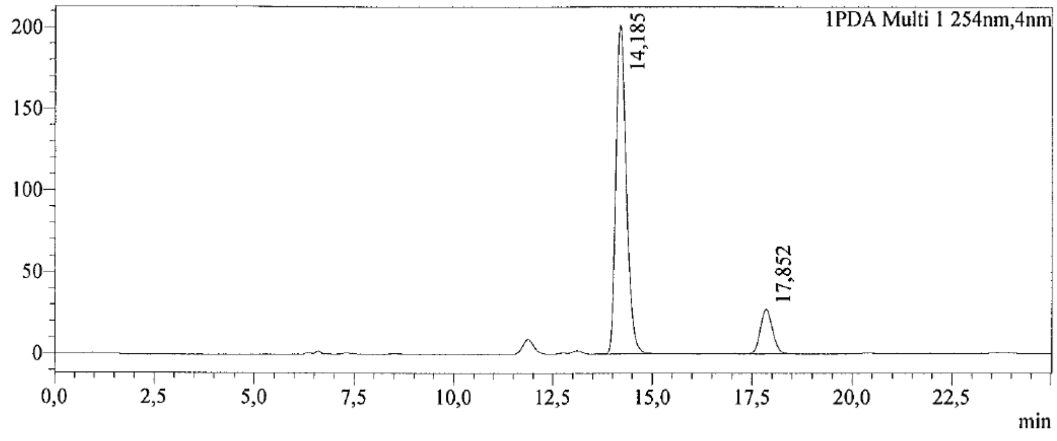

Peak Table

PDA Ch1 254nm

Peak#	Ret. Time	Area	Height	Conc.	Unit	Mark	Name
1	14,185	3695719	201385	86,653		M	
2	17,852	569259	27163	13,347		M	
Total		4264978	228548				

3a formed by **e**•Cu^{2+}

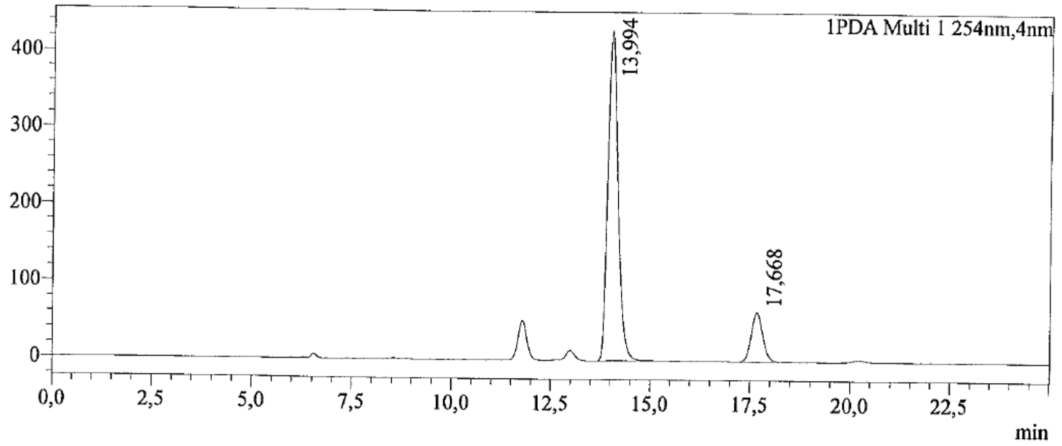

Peak Table

PDA Ch1 254nm

Peak#	Ret. Time	Area	Height	Conc.	Unit	Mark	Name
1	13,994	7660505	429590	85,563		M	
2	17,668	1292527	63981	14,437		M	
Total		8953032	493571				

3a formed by CB [8]• **e**•Cu^{2+}

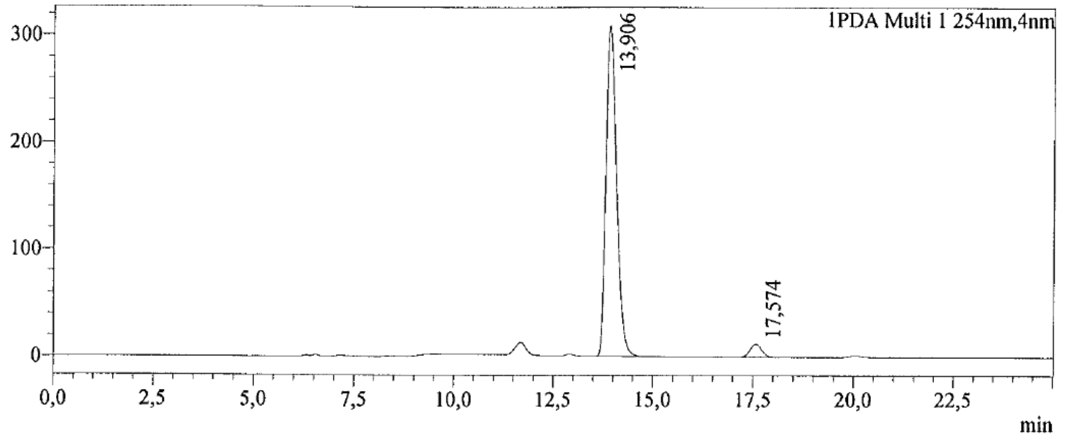

Peak Table

PDA Ch1 254nm

Peak#	Ret. Time	Area	Height	Conc.	Unit	Mark	Name
1	13,906	5685461	308313	95,854		M	
2	17,574	245922	12093	4,146		M	
Total		5931383	320406				

11. References

1. Wang, C.; Jia, G.; Zhou, J.; Li, Y.; Liu, Y.; Lu, S.; Li, C. *Angew. Chem. Int. Ed.* **2012**, *51*, 9352-9355.

2. Huang, W-H; Liu, S.; Isaacs, L. *Cucurbit[n]urils, in: Modern Supramolecular Chemistry: Strategies for Macrocycle Synthesis*, ed. F. Diederich, P. J. Stang and R. R. Tykwinski, Wiley-VCH Verlag GmbH & Co., Weinheim **2008**, *113.*

3.(a) Otto, S.; Boccaletti, G.; Engberts, J. B. F. N. *J. Am. Chem. Soc.* **1998**, *120*, 4238 – 4239; (b) Otto, S.; Engberts, J. B. F. N. *J. Am. Chem. Soc.* **1999**, *121*, 6798 – 6806.

4. Grimme, S.; Antony, J.; Ehrlich, S.; Krieg, H. *J. Chem. Phys.* **2010,** *132*, 154104−154119

5. (a) Miertuš, S.; Scrocco, E; Tomasi, J. *Chem. Phys*. **1981**, *55(1)*, 117-129; (b) Frisch, M. J.; Trucks, G. W.; Schlegel, H. B.; Scuseria, G. E.; Robb, M. A.; Cheeseman, J. R.; Scalmani, G.; Barone, V.; Mennucci, B.; Petersson, G. A.; Nakatsuji, H.; Caricato, M.; Li, X.; Hratchian, H. P.; Izmaylov, A. F.; Bloino, J.; Zheng, G.; Sonnenberg, J. L.; Hada, M.; Ehara, M.; Toyota, K.; Fukuda, R.; Hasegawa, J.; Ishida, M.; Nakajima, T.; Honda, Y.; Kitao, O.; Nakai, H.; Vreven, T.; Montgomery, J. A., Jr.; Peralta, J. E.; Ogliaro, F.; Bearpark, M.; Heyd, J. J.; Brothers, E.; Kudin, K. N.; Staroverov, V. N.; Kobayashi, R.; Normand, J.; Raghavachari, K.; Rendell, A.; Burant, J. C.; Iyengar, S. S.; Tomasi, J.; Cossi, M.; Rega, N.; Millam, M. J.; Klene, M.; Knox, J. E.; Cross, J. B.; Bakken, V.; Adamo, C.; Jaramillo, J.; Gomperts, R.; Stratmann, R. E.; Yazyev, O.; Austin, A. J.; Cammi, R.; Pomelli, C.; Ochterski, J. W.; Martin, R. L.; Morokuma, K.; Zakrzewski, V. G.; Voth, G. A.; Salvador, P.; Dannenberg, J. J.; Dapprich, S.; Daniels, A. D.; Farkas, Ö.; Foresman, J. B.; Ortiz, J. V.; Cioslowski, J.; Fox, D. J. *Gaussian 09, Revision D.01*, Gaussian, Inc., Wallingford CT, **2009**.
